# Remuneration systems of community health workers in India and promoted maternal health outcomes: a cross-sectional study

**DOI:** 10.1186/s12913-019-4883-6

**Published:** 2020-01-20

**Authors:** Hannah J. Koehn, Shenglin Zheng, Robert F. Houser, Corey O’Hara, Beatrice Lorge Rogers

**Affiliations:** 1 Friedman School of Nutrition Science and Policy, Tufts University, 150 Harrison Avenue, Boston, MA 02111 USA; 2 Institute for Health, Health Care Policy and Aging Research, Rutgers, The State University of New Jersey, New Brunswick, NJ 08901 USA; 3 Nevin Scrimshaw International Nutrition Foundation, Boston, MA 02124 USA

**Keywords:** ASHA, AWW, CHW, Performance-based incentives, Maternal and child health

## Abstract

**Background:**

This study assessed the association of remuneration systems of paid-for-performance Accredited Social Health Activists (ASHAs) and salaried Anganwadi workers (AWWs) on seven maternal health outcomes in four states in India: Andhra Pradesh (AP), Chhattisgarh, Odisha (Orissa), and Uttar Pradesh (UP)**.**

**Methods:**

The cross-sectional study surveyed mothers of children aged 6–23 months. A total of 3455 mothers were selected via multistage cluster sampling. The seven health outcomes related to the community health worker (CHW) visits were: institutional delivery, complete immunization, exclusive breastfeeding for six months, timely introduction of complementary feeding, continued breastfeeding during child’s illness, handwashing, and awareness of Nutrition and Health Days (NHDs).

**Results:**

The results varied by state. Mothers who received ASHA visits were significantly less likely to have an institutional delivery, timely introduction of complementary feeding, awareness of Nutrition and Health Days (NHDs), proper handwashing, and exclusive breastfeeding for the first six months in at least one of the four states. Conversely, AWW’s home visits were positively predictive of the following health outcomes in certain states: complete immunization for index child, continued breastfeeding during the child’s illness, handwashing, and awareness of NHDs.

**Conclusions:**

ASHAs’ home visits were not more strongly associated with health outcomes for which they were paid than outcomes for which they were unpaid. AWWs’ home visits were positively associated with awareness of NHDs, and associations varied for other recommended health behaviors. Further research could elucidate the causes for successes and failures of CHW programs in different states of India.

## Background

In India, less than 1% of public spending as a share of GDP is used on health [1]. An acute shortage of health and human resources, infrastructure, and services in rural India is believed to contribute to the country’s high infant and maternal mortality [1]. Community Health Workers (CHWs) act as the link to fill the gap between India’s population and its health care system, especially in terms of maternal and child health services.

Anganwadi workers (AWWs) are local female health workers with at least a 10th grade education, employed under the Integrated Child Development Service (ICDS). AWWs and the other senior level workers, auxiliary nurse-midwives (ANMs), have been the major CHWs in the Indian health care delivery system to meet women and children’s health needs since 1975 [2]. In 2005, the Government of India (GOI) launched the National Rural Health Mission (NRHM) to improve the outreach and coverage of health services. One of its pivotal components was recruiting local females, typically 25–45 years old with a minimum of eight years of education, to train as Accredited Social Health Activists (ASHAs) under the Ministry of Women and Child Development, to supplement the work of AWWs and ANMs [3]. Over the last few years, more than 800,000 ASHAs have been deployed to encourage targeted health behaviors and link the community to public health care to promote maternal and child health in their communities [4]. Each rural village in India is supposed to have an ASHA and an AWW [5].

ASHAs are selected by their communities and receive one month of training. Their role is to provide health promotion, specifically regarding nutrition, sanitation and hygiene, birth preparedness and safe delivery, immunizations, breastfeeding, complementary feeding, and prevention of common infections. While they are considered unpaid volunteers, ASHAs receive performance-based incentives for facilitating institutional deliveries and immunizations of children in addition to receiving compensation for their training days and attending monthly meetings. ASHAs receive approximately $10 for facilitating an institutional delivery and $3 for facilitating a child’s immunization session, though compensation varies by state [6, 7].

AWWs are also selected by the community. They receive two to three months of training, and their role is to provide health information, medicine, and nutritional supplementation to children under six years old, adolescent girls, and pregnant and lactating women [5, 6]. AWWs receive a monthly stipend of approximately $25 and qualify for a government life insurance scheme [6]. There are some conflicting results on the financial incentives received by ASHAs. A mixed-method study of ASHAs in Orissa (now Odisha) found that ASHAs were more motivated by the social recognition, a sense of social responsibility, and self-efficacy to perform their responsibilities than by their incentives [8]. Other researchers have shown that the performance-based incentives may be a key factor to an ASHAs’ performance [9], which can lead to ASHAs focusing more on the activities for which they are paid than unfunded activities [10].

However, there is scarce evidence of the effects of home visits conducted by the two types of workers on maternal and children health outcomes. The purpose of this study is to determine, using data from four Indian states, 1) whether ASHAs’ home visits are more predictive of institutional deliveries and children’s complete immunization, for which they are paid, than of five unpaid, but important health practices: exclusive breastfeeding for the first six months of the child’s life, timely introduction of complementary feeding, continued breastfeeding during the child’s illness, proper handwashing, and awareness of Nutrition and Health Days (NHDs); 2) whether visits from AWWs are more predictive of certain health outcomes than other health outcomes.

## Methods

### Data

This analysis used the secondary quantitative survey data from the 2011 follow-up of the 2009 CARE endline evaluation of USAID’s Food for Peace (FFP) projects in four states in India: Andhra Pradesh (AP), Chhattisgarh, Odisha, and Uttar Pradesh (UP) [11]. The 2011 survey used multistage cluster sampling using random selection with probability proportional to size. Randomly selected *Anganwadi* Centers (AWC) from a list of centers in each state in districts with a CARE program were used to identify catchment areas. The study team conducted a census in each selected catchment area and identified households with children under two years of age. From the list of households, children aged 0–5 months and 6–23 months were randomly selected for the 2011 survey. This study used data from the mothers of 6–23-month-old children (*N* = 3455).

### Independent variables

The independent variables were the same for each of the seven outcomes. CHW home visits were based on responses to the survey question, “Has a *(specify the type of CHW)* met you at home in the last 1-month to talk to you about the care and feeding of your child?” Two binary CHW home visit options were analyzed in the logistic regression, ASHAs and AWWs.

- *ASHAs* represents mothers who reported they were visited by an ASHA and could also have been visited by any other type of CHWs to talk about the care and feeding of her child in the last one month.^1^
- *AWWs* represents mothers who reported they were visited by an AWW and could also have been visited by any other type of CHWs to talk about the care and feeding of her child in the last one month.^2^

### Dependent variables

ASHAs receive performance-based incentives, and though they are expected to promote the following seven outcomes, they are mainly paid for assisting the mother in delivering at an institutional facility (Outcome 1) and for ensuring that her child has completed the correct immunizations for her/his age (Outcome 2). AWWs are paid a salary and do not receive incentives for promoting certain health outcomes over any other health outcomes.

- *Outcome 1: Institutional Delivery:* Defined as a mother having delivered the index child in a sub center, community health center (CHC), primary care hospital (PCH), government hospital, or in a private clinic/hospital.
- *Outcome 2: Complete immunization:* Based on India’s National Immunization Schedule. Index children younger than nine months of age were considered to have complete immunization if their mothers reported or showed an immunization card inclusive of BCG, OPV, OPV1, OPV2, OPV3, DPT1, DPT2, DPT3, HepB1, HepB2, and HepB3. An index child aged nine to twenty-three months of age was considered to have complete immunization if s/he had all previously mentioned immunizations plus the immunization for measles.
- *Outcome 3: Exclusive Breastfeeding for Six Months:* Mothers who reported that they did not give any liquid, semi-solid, or solid food other than medicine and breastmilk to their child in the first six months of life were considered to have exclusively breastfed.
- *Outcome 4: Timely Introduction of Complementary Feeding Starting from 6 to 9 Months:* A mother was considered to have timely introduction of complementary feeding if she began giving the index child any liquids (other than breastmilk), semi-solids or solid foods between six and nine months of age.^3^
- *Outcome 5: Continued Breastfeeding during Child’s Illness:* Mothers who had a child who had diarrhea in the past two weeks and continued to breastfeed the same or more often than usual.
- *Outcome 6: Handwashing:* A mother was considered to have correct handwashing practices if she reported that she usually washes her hands with soap before cooking food, before eating food, and after defecation.
- *Outcome 7: Awareness of Nutrition and Health Days (NHDs):* Mothers self-reported if they were aware of NHDs held at the AWC.^4^

### Covariates

The covariates used in this study were demographic characteristics of mothers and their households and other CHW visits. The demographic covariates included: house type (kuchcha, semi-pucca, pucca), region type (urban, rural, tribal), sex of the index child, total household size, the ratio of the number of children under five years of age to the number of adult females in the household (an indicator of demands on the mother’s time and attention), the highest education level completed by any adult in the household, age of the mother, and paid employment status (yes/no) of the mother. ASHAs and AWWs serve the same catchment area and beneficiary population and are expected to be comparable.

In addition, other CHWs and multiple visits from different types of CHW were considered as covariates in this study. Other CHWs represents mothers who reported they were visited by either an ANM or a Lady Health Visitor (LHV) to talk about the care and feeding of her child in the last one month. Multiple Visits represents a mother who reported that she received at least two home visits from different types of CHWs (ASHAs, AWWs, ANMs, LHVs) to talk about the care and feeding of her child in the last one month.

### Analysis

The descriptive data are shown as N(%) or Mean ± SE. Multivariate logistic regression models were tested for each of the seven outcomes adjusting for the complex sampling design using survey weights from the 2011 study. All models included the same two independent variables and covariates to control for demographic characteristics of mothers and their households and for other CHW visits. There was no multicollinearity among the independent variables. Odds ratios were reported for each of the independent variables along with 95% confidence intervals (CI). Statistical significance was determined as a *p*-value ≤0.05, *p*-value ≤0.01, and *p*-value ≤0.001. The analyses were conducted using Stata/MP 14.1.

## Results

### Description of study sample

Table 1 presents the demographic and socioeconomic characteristics of the household and the mothers. From Table 2, the percentage of households reporting ASHA home visits was lower than that of AWWs or other CHWs in all four states and was lowest in AP (4.72%). Only in Odisha did the percentage of households receiving ASHA home visits reach 24.50%. AP had the highest rate of AWW and other CHW home visits (53.94 and 54.00%, respectively) compared to other states. Home visits conducted by AWWs were more common than other types of health care workers in all four states, with percentages from 31.20 to 53.94%. However, the percentage of no CHW visits was also high, ranging from 34.19 to 51.65%.

**Table 1:** Mother and Household Demographics, Overall and in Four States^a^

	Overall	Andhra Pradesh	Chhattisgarh	Odisha	Uttar Pradesh
House type^b^					
Kuchcha	1617 (48.56)	140 (14.74)	497 (62.61)	655 (73.99)	325 (40.19)
Semi-Pucca	1117 (31.37)	73 (49.43)	190 (23.2)	96 (10.72)	358 (44.58)
Pucca	720 (30.06)	337 (35.83)	122 (14.19)	136 (15.29)	125 (15.23)
Region					
Urban	531 (14.63)	234 (24.15)	104 (12.12)	60 (6.97)	133 (16.1)
Rural	937 (26.87)	252 (26.77)	352 (44.61)	254 (25.8)	79 (10)
Tribal	1987 (58.5)	464 (49.08)	353 (43.27)	573 (67.24)	597 (73.9)
Caste/Tribe					
Scheduled Tribe	886 (20.72)	138 (17.48)	297 (39.56)	446 (53.21)	5 (0.88)
Scheduled Caste	665 (20.98)	160 (20.69)	115 (14.1)	118 (13.85)	272 (38.06)
Other Backward Caste	1364 (42.06)	433 (53.94)	305 (39.56)	285 (30.22)	341 (48.19)
Other Caste	241 (7.24)	70 (7.89)	56 (6.781)	25 (2.71)	90 (12.87)
Sex of Index Child					
Male	1743 (50.39)	514 (54.35)	408 (49.67)	413 (47.04)	408 (50.86)
Female	1712 (49.61)	436 (45.65)	401 (50.33)	474 (52.96)	401 (49.14)
Care Ratio^c^	1.11 ± 0.01	0.77 ± 0.02	1.19 ± 0.03	1.18 ± 0.02	1.30 ± 0.03
	(*N* = 3323)	(*N* = 848)	(*N* = 802)	(*N* = 885)	(*N* = 788)
Household Size	5.06 ± 0.07	3.28 ± 0.05	5.87 ± 0.08	5.22 ± 0.06	5.80 ± 0.07
	(*N* = 3454)	(*N* = 950)	(*N* = 809)	(*N* = 887)	(*N* = 808)
Religion of the Head of the Household					
Hindu	3705 (89.2)	840 (88.43)	648 (80.74)	856 (96.51)	731 (90.37)
Muslim	126 (3.51)	26 (2.65)	26 (2.68)	0	74 (9.25)
Tribal	136 (4.17)	15 (1.75)	112 (13.9)	9 (1.26)	0
Others	117 (3.12)	69 (7.17)	23 (2.68)	22 (2.23)	3 (0.39)
Highest Education Completed by any Adult in the Household^d^					
None	1161 (26.06)	267 (28.99)	150 (18.37)	257 (30.5)	208 (25.87)
1–4 years	157 (4.72)	20 (2.13)	39 (4.78)	81 (9.33)	17 (2.10)
5–10 years	1684 (48.79)	425 (44.55)	437 (54.38)	412 (45.91)	410 (50.69)
11–12 years	340 (9.57)	100 (10.06)	97 (12.09)	61 (6.34)	82 (10.14)
13+ years	392 (10.87)	138 (14.26)	86 (10.38)	76 (7.92)	92 (11.21)
Age of Mother	25.32 ± 0.09	24.12 ± 0.10	24.59 ± 0.14	25.97 ± 0.15	26.55 ± 0.15
	(*N* = 3455)	(*N* = 950)	(*N* = 809)	(*N* = 887)	(*N* = 809)
Mother’s Paid Employment Status^e^					
No	1653 (46.8)	445 (46.12)	366 (44.19)	507 (54.91)	335 (40.97)
Yes	1800 (53.2)	505 (53.88)	443 (55.81)	379 (45.09)	473 (59.03)

a. The data are shown as N (%) except care ratio, household size, and age of the mother presented as Mean ± SE

b. House types defined as Pucca house, Semi-Pucca house and Kuchcha house. A pucca house is made with high quality materials, including roofs, floors, and walls. A kuchcha house is made with low quality materials such as mud and thatch

c. Care ratio is the number of children under 5 years of age divided by the number of adult females in the household

d. An adult defined as a person aged 16 or older

**Table 2:** Percentage of Home Visits by Types of CHWs & Health Practices, Overall and in Four States

	Overall *N* (%)	Andhra Pradesh N (%)	Chhattisgarh *N* (%)	Odisha *N* (%)	Uttar Pradesh *N* (%)
Home Visits					
ASHAs	375 (12.17)	42 (4.72)	54 (7.79)	206 (24.50)	73 (9.51)
AWWs	1434 (41.07)	520 (53.94)	252 (31.20)	337 (38.77)	325 (40.50)
Other CHWs	1004 (27.99)	520 (54.00)	99 (11.64)	179 (21.18)	206 (25.62)
Multiple Visits	936 (27.83)	426 (45.52)	99 (13.56)	228 (27.21)	183 (23.55)
Health Practices					
Institutional Delivery	2484 (70.75)	899 (94.39)	410 (50.22)	630 (69.43)	556 (68.88)
Complete Immunization	256 (7.78)	170 (20.35)	22 (3.32)	8 (0.78)	56 (7.43)
Exclusive Breastfeeding for First 6 Months	1639 (46.39)	700 (73.54)	159 (19.62)	598 (66.97)	182 (22.43)
Timely Introduction of Complementary Feeding	2655 (78.70)	608 (68.33)	686 (85.38)	731 (81.63)	630 (78.53)
Continued Breastfeeding During Child’s Illness	899 (66.41)	253 (77.83)	319 (66.83)	210 (68.80)	117 (48.73)
Handwashing	1940 (55.57)	484 (50.68)	435 (52.85)	369 (40.35)	652 (80.73)
Awareness of NHDs	1712 (49.43)	599 (63.44)	470 (58.36)	438 (49.54)	205 (25.62)

For the seven health outcomes assessed in this study, the adoption rates of practices varied by state. Overall, more than 50% of mothers achieved four out of the seven health outcomes, with the lowest adoption rate for complete immunization (7.78%) (Table 2). Among the health outcomes, institutional delivery was the second most adopted outcome overall (70.75%), being particularly high in AP (94.39%). AP also had the highest rate of exclusive breastfeeding for the first six months (73.54%), continued breastfeeding during the child’s illness (77.83%), and awareness of NHDs (63.44%). Chhattisgarh had the highest rate of adopting timely complementary feeding (85.38%), and UP had the highest rate of handwashing (80.73%).

#### Multivariate regression analyses

#### Institutional delivery

A visit by any one type of CHW or multiple CHWs did not have a significant effect on the odds of a mother having an institutional delivery among the four states, with the exception of ASHAs in AP. Contrary to the study hypothesis, in AP, holding all else equal in the logistic regression, a mother visited by an ASHA was 96% less likely to have an institutional delivery than a mother not having had a visit from an ASHA (OR = 0.04, 95% CI = 0.1–0.16)^5^ (Table 3).

**Table 3:** Adjusted Odds Ratios of Seven Outcomes in Four States in India, by Type of CHW Visit^ab^

	Andhra Pradesh		Chhattisgarh		Odisha		Uttar Pradesh	
	ASHAs	AWWs	ASHAs	AWWs	ASHAs	AWWs	ASHAs	AWWs
	Adjusted OR (95% CI)		Adjusted OR (95% CI)		Adjusted OR (95% CI)		Adjusted OR (95% CI)	
Institutional Delivery	*n* = 698		*n* = 664		*n* = 845		*n* = 665	
	0.04***	0.31	1.61	0.81	0.96	1.26	1.31	0.96
	(0.01–0.16)	(0.07–1.46)	(0.75–3.43)	(0.48–1.36)	(0.60–1.54)	(0.71–2.26)	(0.66–2.63)	(0.55–1.68)
Complete Immunization	*n* = 637		*n* = 488		*n* = 113		*n* = 622	
	1.79	0.57	1.00	4.82**	0.77	1.80	1.74	1.33
	(0.48–6.61)	(0.21–1.54)	(1.00–1.00)	(1.50–15.46)	(0.10–6.10)	(0.02–151.81)	(0.66–4.60)	(0.62–2.83)
Exclusive Breastfeeding for First 6 Months	*n* = 698		*n* = 664		*n* = 870		*n* = 665	
	0.62	1.71	0.23*	0.68	0.55**	1.04	0.59	1.66
	(0.18–2.16)	(0.87–3.38)	(0.06–0.84)	(0.36–1.28)	(0.36–0.84)	(0.64–1.67)	(0.24–1.47)	(0.86–3.22)
Timely Introduction of Complementary Feeding	*n* = 652		*n* = 663		*n* = 869		*n* = 658	
	0.17***	1.37	2.20	0.61	0.64	1.19	0.96	1.05
	(0.05–0.57)	(0.70–2.67)	(0.59–8.18)	(0.34–1.10)	(0.37–1.12)	(0.61–2.34)	(0.45–2.05)	(0.64–1.74)
Continued Breastfeeding During Child’s Illness	*n* = 223		*n* = 389		*n* = 300		*n* = 197	
	1.00	0.33	0.73	2.06*	0.62	0.93	0.57	0.98
	(1.00–1.00)	(0.09–1.27)	(0.27–1.93)	(1.10–3.85)	(0.32–1.21)	(0.41–2.13)	(0.16–2.00)	(0.49–1.95)
Handwashing	*n* = 698		*n* = 664		*n* = 870		*n* = 665	
	1.41	0.40*	1.37	1.35	1.29	1.65*	1.66	0.86
	(0.22–8.93)	(0.17–0.94)	(0.82–2.29)	(0.82–2.22)	(0.81–2.08)	(1.05–2.60)	(0.50–5.54)	(0.52–1.43)
Awareness of NHDs	*n* = 698		*n* = 664		*n* = 870		*n* = 665	
	0.11***	9.41***	1.37	1.78*	1.74*	2.50***	0.38	2.24**
	(0.03–0.37)	(3.85–23.00)	(0.75–2.50)	(1.06–2.96)	(1.14–2.66)	(1.61–3.90)	(0.11–1.28)	(1.24–4.04)

a. the models were adjusted by multiple CHW visits, other CHW’s visits, house type, region type (urban, rural, tribal), sex of index child, total household size, the ratio of children under five years of age to the number of adult females in the household, highest education level completed by any adult in the household, mother’s age, and mother’s employment status

b. ASHAs and AWWs are binary variables. ASHAs represents a mother who was visited by an ASHA (yes/no). AWWs represents a mother who was visited by an AWW (yes/no)

*p ≤ 0.05, **p ≤ 0.01, ***p ≤ 0.001.

#### Complete immunizations

Among the CHWs across the four states, only visits from AWWs significantly predicted complete immunizations for the index child. Table 3 illustrates that in Chhattisgarh, the odds of having a child between the ages of 6 and 23 months completely immunized was over four times greater (OR = 4.82, 95% CI = 1.50–15.46)^6^ if the mother received a visit from an AWW, while ASHA visits had no significant effect.

#### Exclusive breastfeeding for first six months

In both Chhattisgarh and Odisha, visits conducted by ASHAs had a statistically significant negative association with reported exclusive breastfeeding for the first six months of a child’s life. In Chhattisgarh, all else equal, mothers were significantly less likely to report that they exclusively breastfed their child for the first six months of life if they had a visit from an ASHA (OR = 0.23, 95% CI = 0.06–0.84). In Odisha, mothers who had been visited by an ASHA were 45% less likely to report exclusive breastfeeding (OR = 0.55, 95% CI = 0.36–0.84).

#### Timely introduction of complementary feeding

In AP having an ASHA visit was associated with significantly lower odds of a mother introducing complementary feeding during the recommended period; there was no significant effect in any other state. All else equal, the odds of introducing liquids (other than breast milk), solids, or semi-solids into the index child’s diet between six and nine months (the behavior recommended by the CHWs) was 83% less likely among mothers who had a visit from an ASHA (OR = 0.17, 95% CI = 0.05–0.57)^7^ (Table 3).

#### Continued breastfeeding during Child’s illness

In Chhattisgarh, mothers who received a visit from an AWW were more than twice as likely to report continued breastfeeding during their child’s illness (OR = 2.06, 95% CI = 1.10–3.85). This sample only included mothers who had reported that their child had diarrhea within the past two weeks, and in Chhattisgarh that included 389 out of 664 mothers surveyed. While it is encouraging that AWW visits had a significant positive association with continued breastfeeding during illness, it is alarming that over 50% of women in the sample for this state reported their children having diarrhea within the past two weeks.

#### Handwashing

In Odisha, all else equal, a mother was slightly more likely to follow proper handwashing techniques if she was visited by an AWW (OR = 1.65, 95% CI = 1.05–2.60) than were those not visited by an AWW; however, in AP, the result was the opposite (OR = 0.40, 95% CI = 0.17–0.94) (Table 3).

#### Awareness of NHDs

In all four states, mothers who received a visit from an AWW were significantly more likely to report being aware of NHDs held at their local AWC (Table 3). In Odisha, mothers were also 74% more likely to report awareness of NHDs if they received a visit from an ASHA (OR = 1.74, 95% CI = 1.14–2.66), but in AP, mothers who received a visit from an ASHA were much less likely to be aware of NHDs (OR = 0.11, 95% CI = 0.03–0.37).

## Discussion

This analysis shows that visits by ASHAs did not demonstrate a stronger relationship with the health outcomes for which they were paid (institutional delivery and complete immunizations) than with outcomes for which they were not explicitly paid (exclusive breastfeeding for the first six months, timely introduction of complementary feeding, continued breastfeeding during the child’s illness, handwashing, and awareness of NHDs). With few exceptions, ASHA visits did not show a significant positive impact on these health behaviors, and in some instances showed a negative association. The analysis also demonstrates that visits from AWWs were more predictive of mothers adopting certain health behaviors (complete immunizations for the index child and continued breastfeeding during the child’s illness in Chhattisgarh, handwashing in Odisha, and awareness of NHDs in all four states). Visits by other CHWs and multiple visits by different types of CHWs were not predictive of any of the health outcomes in any of the four states. The study also ran a model (data not shown) with an interaction term between visits from ASHAs and AWWs, to see if visits from one worker reinforced or otherwise affected the effect of visits from the other, but no significant effects were observed.

Table 3 shows that a mother in AP is less likely to have an institutional delivery after being visited by an ASHA. The reported odds ratio may be misleading, however. Only 42 mothers in AP were visited by ASHAs – a very small number compared to other types of CHW visits (AWWs 520 and Other CHWs 520) – and these mothers may be anomalous in other, unmeasured ways. Of those 42 mothers, over 71% had an institutional delivery. The same issue of minimal ASHA visits in AP applies to the statistically significant but possibly misleading results for having an ASHA worker visit reduce the odds of a mother introducing complementary feeding as recommended and being aware of NHDs.

Similarly, the impact of a visit from an AWW in Chhattisgarh may not be as extreme as the odds ratio implies. A visit from an AWW statistically increased the likelihood of an index child being completely immunized, but only 22 children in the state had complete immunizations out of 692, suggesting that the positive impact of an AWW visit is overstated in the statistical results, and that CHW visits, in fact, did not achieve the goal of universal complete immunization.

Some studies have shown that more than 60% (689/1141) of institutional deliveries in India can be attributed to the motivation of ASHAs [4]. Similarly, a recent study showed that exposure to ASHA services was associated with a 28% increase in facility births [13]. However, Wagner and his colleagues did not find a significant relationship between ASHA placement in a community and institutional delivery [14]. Rather than motivation, exposure to services, or ASHA placement, our study used ASHA home visits as a primary independent variable and found no positive association with institutional deliveries, which is likely a key reason for the difference in findings compared to other research.

Bellows and his colleagues reported that ASHAs focused more on the health practices for which they were paid at the expense of other important but unpaid activities [10], which is not supported by the results of this analysis. A qualitative study by Saprii L. and colleagues in 2015 found that ASHAs relied on the incentives provided from institutional deliveries and referrals of pregnancy cases, but that other activities were poorly incentivized [15]. However, ASHAs in remote villages found it more difficult to rely on the incentives provided from pregnancy referrals and institutional deliveries because there were too few pregnancy cases in their communities [15]. In 2010, Scott K. and Shanker S. also reported that ASHAs were limited by the performance-based payments and delayed incentives [16]. Some ASHAs have campaigned to change their payment method to a regular salary-based system rather than the unstable performance-based incentives, but this has not been approved by the NRHM [17]. Differences in the compensation system within states, variability in pregnancy cases within communities, and ability to rely on the incentives provided to ASHAs were not controlled for in this analysis but may all affect the results presented above.

Another explanation for the statistically insignificant association between ASHA visits and the promoted maternal health outcomes may be that the ASHA program was established in 2005, whereas the AWW program has been in place since 1975. Within the six years between the start of the ASHA program and the survey reported here, the GOI may not have fully developed the capability to support the ASHAs. Consistent with this, Paul et al. also stated that the Indian primary health care system lacks sufficient ASHA training, supervision, and monitoring, both nationally and at the state level [18]. Without sufficient support from the government and health system, ASHA workers may not have received the necessary resources for their work, which could account for the low rates of ASHA home visits revealed in this analysis.

Given the low percentage of households that received an ASHA visit, it is possible that these households have other characteristics associated with the outcomes measured. Although we controlled for possible confounders in multivariate analysis, unobserved or unmeasured differences between households that received an ASHA visit and those that did not could have contributed to the results reported here.

Future studies could employ a cluster randomized design in which workers are assigned to be paid according to one or the other compensation method and evaluate whether a hybrid compensation model may achieve superior positive impacts on maternal and child health outcomes in India. CHWs could be salaried and also have the opportunity to receive performance-based incentives such as those currently given to ASHAs. The stability of a salaried remuneration system could attract the most qualified CHWs, while offering the performance-based incentives may encourage the CHWs to prioritize key outcomes.

### Study limitations and implications

The Tufts University study team replicated the FFP projects’ quantitative endline evaluation surveys in 2011 for CARE. The researchers consequently did not have control over the questionnaire design and were unable to control for useful factors such as information about home visits, state-level incentives, or adequacy of trainings of AWWs and ASHAs. The survey question, “Has a *(specify the type of CHW)* met you at home in the last 1-month to talk to you about the care and feeding of your child?” may also have been overly specific. It is possible that some mothers did not report a visit by a CHW because the CHW who visited them did not discuss the care and feeding of their children, but may have discussed other important health outcomes, such as proper handwashing practices. Finally, because analyzing outcomes based on home visits from different types of CHWs was the purpose of our analysis, this study could have benefited from having data on the number of home visits by various CHWs.

Due to the diverse results across states in this analysis, we suggest further research on this topic that includes variables related to the implementation of the ASHA program, inclusive of incentives being paid out by state and the number of active ASHAs, in order to identify possible reasons why ASHA visits are lower in some states and higher in others. It would be useful to identify the factors underlying the successes and challenges of the various CHWs’ performance and compare the AWW and ASHA programs order to provide recommendations to improve the impact of the ASHAs on the health and nutrition outcomes of their beneficiaries.

## Conclusions

This study suggests that the pay-for-performance model of the ASHAs did not show greater impact on specific health behaviors for which they are paid. However, AWWs had a consistent positive association with awareness of NHDs in four states, and associations with other recommended health outcomes varied by state.

## Data Availability

The datasets used and/or analyzed during the current study are available from the corresponding author on reasonable request.

## Abbreviations

ANM: Auxiliary Nurse-Midwives
ASHA: Accredited Social Health Activists
AWW: Anganwadi workers
CHW: Community Health Worker
GOI: Government of India
ICDS: Integrated Child Development Service
NHD: Nutrition and Health Days
NRHM: National Rural Health Mission

## Footnotes

1 Depending on the state, 60–70% of mothers who were visited by an ASHA were also visited by another CHW.

2 Depending on the state, 45–82% of mothers who were visited by an AWW were also visited by another CHW.

3 The World Health Organization recommends the introduction of complementary food from 6 months onwards, but CARE’s evaluation asked about the introduction of complementary food at 6–9 months of age [12].

4 NHDs are organized monthly at the AWC serving the communities. At the NHDs, children are weighed, their health cards are checked, appropriate immunizations are given, and take-home rations are provided. NHDs are attended by AWWs and ASHAs.

5 In AP, 42 mothers were visited by an ASHA.

6 In Chhattisgarh, 22 children had completed immunizations.

7 In AP, 42 mothers were visited by an ASHA.
